# Characterizing morphology of *Egregia menziesii* (Laminariales) in California over 2 centuries using historical and contemporary herbarium specimens

**DOI:** 10.1111/jpy.70126

**Published:** 2026-01-20

**Authors:** Adi Khen, Kai M. Moore, Siobhan A. Braybrook, Peter S. Vroom, Kathy Ann Miller, Jennifer E. Smith

**Affiliations:** ^1^ Center for Marine Biodiversity and Conservation, Scripps Institution of Oceanography University of California San Diego La Jolla California USA; ^2^ Department of Molecular, Cell and Developmental Biology University of California Los Angeles Los Angeles California USA; ^3^ San Diego Natural History Museum San Diego California USA; ^4^ University Herbarium, University of California at Berkeley Berkeley California USA

**Keywords:** feather boa kelp, herbarium, image analysis, intraspecific variability, macroalgae, morphology, natural history

## Abstract

The canopy‐forming feather boa kelp *Egregia menziesii* exhibits remarkable morphological variability across its geographic range. Regional morphotypes of *Egregia* were once considered separate species, but they were not determined to be genetically distinct; instead, their morphology was thought to reflect local physical or environmental conditions. Although morphological variation in *Egregia* has long been observed and was previously characterized through field surveys in the early 2000s, we revisited this topic using digital morphometrics (i.e., image analysis) of 1624 macroalgal herbarium specimens from California dating back to the 19th century. We observed that the morphology of *Egregia* (rachis texture, lateral blade shape, and blade or pneumatocyst density) varied along a latitudinal gradient and could be predicted by seawater temperature and wave height. We also identified some region‐specific morphological changes in recent decades. Further, the monthly presence or absence of sporophylls in southern‐region specimens provided preliminary evidence into the reproductive phenology of *Egregia*. Herbarium collections are invaluable for studying patterns in morphology because they showcase inter‐ and intraspecific variability and establish a baseline for comparison through time. Integrating natural historical and contemporary data will be critical for understanding and predicting future trends in the context of ocean warming.

**Abbreviations**
ITS: internal transcribed spacer
MFA: multiple factor analysis
SST: sea surface temperature

## INTRODUCTION

### Taxonomic history

*Egregia menziesii* (Turn.) Aresch., as generally understood, is common along the whole coast and presents a number of very distinct and puzzling forms. (Setchell, 1896, p. 43)

The feather boa kelp *Egregia menziesii* is a canopy‐forming foundation species that grows in intertidal and subtidal habitats along the Pacific coast of North America. Setchell (1893) described a northern form with a tough, tuberculate rachis and terminal blade that matched the original description of *Fucus menziesii* (type locality: Vancouver Island, British Columbia, Canada; Turner, 1808). Setchell noted that specimens south of Point Conception, California were characterized by a smooth rachis and a diversity of lateral blade shapes, many dissected. He proposed the name *E. laevigata* (lectotype locality: San Pedro, Los Angeles County, California) for the southern form, noting that both forms occurred in San Luis Obispo and Santa Cruz counties, with the southern form absent from San Francisco and the northern form absent from San Pedro (Setchell, 1896). Setchell later described *E. laevigata* forma *borealis* (type locality: Carmel Bay, Monterey County) to accommodate *laevigata*‐like forms in Central California counties.

Silva (1957), following an intensive field study of *Egregia* on the mainland between Carmel and Santa Barbara and throughout the California Channel Islands, observed that “the two species are seen to be far less sharply delimited than was previously believed” (p. 45) and suggested the possibility of hybridization. Silva described an island subspecies from the northern Channel Islands, *E. menziesii* subsp. *insularis* with a tough tuberculate rachis but highly dissected older lateral blades, unlike the northern form. He also described *E. laevigata* subsp. *borealis*, based on *E. laevigata* forma *borealis* as occurring from Gaviota to Santa Cruz, with a smooth, brittle rachis, occasional short tubercles, and blades like those of *E. laevigata* but widely spaced and never dissected. He changed the status of Setchell's forma to subspecies to assert that the morphology of this taxon was correlated with geography. Silva concluded: “A case might well be argued for recognizing in *Egregia* only one species comprised of several subspecies. It seems of greater taxonomic usefulness, however, to continue the recognition of two species” (p. 46).

Chapman (1961) claimed that “there is little difficulty in distinguishing the three major species, especially since they are geographically distinct” (p. 39), referring to a proposed regional distribution in which *Egregia menziesii* would be located in the north from Alaska to Point Conception, California; *E. laevigata* between Point Conception and Ensenada, Mexico, and *E. planifolia* south of San Diego, California, into Baja California, Mexico. Chapman compared shallow (i.e., intertidal) and deep‐water (>3 m depth) forms of *E. laevigata*, noting that subtidal thalli grew to greater lengths and had more pneumatocysts, fewer epiphytes, and blades eroded at the edges, whereas intertidal thalli were shorter overall, sometimes lacked pneumatocysts, and their blades were highly dissected or branched (Chapman, 1961).

Abbott and Hollenberg (1976) recognized a single species, *Egregia menziesii*, with characters mapped to northern (Alaska to Cape Mendocino; *E. menziesii*), southern (Los Angeles to Baja California, Mexico; *E. laevigata*), and central populations (Cape Mendocino to Ventura counties and the Channel Islands), which “possess every possible combination of features observed in the geographic extremes and include a few unique vegetative and reproductive morphologies” (p. 244). Stewart (1991, p. 54) commented on “the extensive morphological variability observed in different parts of the same plant, among plants in the same area, and throughout the range of the species.”

### Modern understanding

Currently, *Egregia* is considered monospecific, with *E. menziesii* being the only taxonomically accepted species based upon molecular data from the nuclear internal transcribed spacer (ITS) rRNA regions ITS1 and ITS2 (Henkel et al., 2007). *E. menziesii* individuals sampled from populations at 12 sites from Oregon to southern California, covering a wide range of morphological variation, were grouped into one phylogenetic clade (Henkel et al., 2007). However, two regional morphotypes are still recognized: Northern‐type *Egregia* are characterized by a highly branched thallus, papillated rachis, and more compact lateral blades, and southern‐type *Egregia* have smooth rachis and larger, widely spaced blades (Abbott & Hollenberg, 1976). Northern morphotypes produce less drag (Friedland & Denny, 1995) and have a higher breaking strength (Blanchette et al., 2002), which is advantageous in intense wave energy, whereas the larger blade surface area in southern morphotypes presumably helps to maximize nutrient uptake in warmer, more nutrient‐limited waters (see Rosenberg & Ramus, 1984). In a laboratory study on tissue mechanics and hydrodynamic adaptation, juvenile *E. menziesii* cultured at a high water velocity for 6–10 weeks were stronger, stiffer, and thicker than those cultured with minimal water motion (Kraemer & Chapman, 1991a). In reciprocal transplant experiments of adult *E. menziesii* on the northern and southern sides of Point Conception, individuals performed better in terms of growth and survival at sites with conditions typical of their native region after 5 months (Blanchette et al., 2002). Thus, morphological differences in *E. menziesii* are likely responses to local conditions, and the vast phenotypic plasticity of *E. menziesii* is tied to environmental rather than genetic factors.

### Objectives and justification

To explore relationships between geography, environment, and morphology, the main objective for this study was to characterize the morphological variability of *Egregia menziesii* across space and time using images of historical and contemporary herbarium specimens. We focused on California because it has both northern and southern morphotypes as well as an extensive digitized herbarium record dating back almost 2 centuries. We classified or quantified morphological traits with more categories and nuance than were previously used—including additional lateral blade shapes, their co‐occurrence with rachis texture, and blade or pneumatocyst density—and we investigated whether morphology was correlated with latitude, seawater temperature, wave height, and/or upwelling (as a proxy for nutrients). We also described the reproductive phenology of *E. menziesii* in southern California through the monthly presence or absence of sporophylls seen in herbarium specimens. We were primarily interested in comparing our herbarium‐based morphological characterization of *E. menziesii* to the characterizations reported in the literature and in determining whether past and present herbarium records might reveal morphological changes through time. We approached this with a unique perspective: reexamining historical specimens through the modern understanding that they represent a single species, while also broadening the existing framework for its morphological description.

## MATERIALS AND METHODS

### Data collection

We downloaded images and collection metadata for all digitized herbarium specimens of *Egregia menziesii* in California from the Algae Herbarium Portal (2025) (https://macroalgae.org/portal/), the University Herbarium at UC Berkeley (2025) (https://ucjeps.berkeley.edu/), and the newly established Ellen Browning Scripps Herbarium Collection at Scripps Institution of Oceanography (Smith & Khen, 2025). We then verified, cleaned, reformatted, and compiled the datasets. After excluding all juvenile individuals, we had a total sample size of 1624 adult specimens, spanning from July 1828 to February 2025. Our dataset included information such as the collection date; the city and county of collection, which were binned into regions (with the five northern coastal counties comprising northern California, the five southern coastal counties comprising southern California, and the five counties in between comprising central California); geographic coordinates of collection; and whether the specimen was collected intertidally or subtidally, when noted. We used the National Oceanic and Atmospheric Administration's monthly global Extended Reconstructed Sea Surface Temperature Record (NOAA ERSST version 5; Huang et al., 2017) to extract average annual sea surface temperatures (SSTs) for years and locations in which specimens were collected. For a subset of specimens (*n* = 194) collected since the late 1980s for which other environmental time series data were available, we also incorporated wave height and upwelling values averaged yearly by location. Specifically, we used NOAA's WAVEWATCH III Global Wave Model (Tolman, 2009), which calculates the significant height of combined wind waves and swell, and the Biologically Effective Upwelling Transport Index (BEUTI; Jacox et al., 2018), which estimates the amount of nitrate upwelled or downwelled near the coast.

### Morphological characterization

**FIGURE 1:**
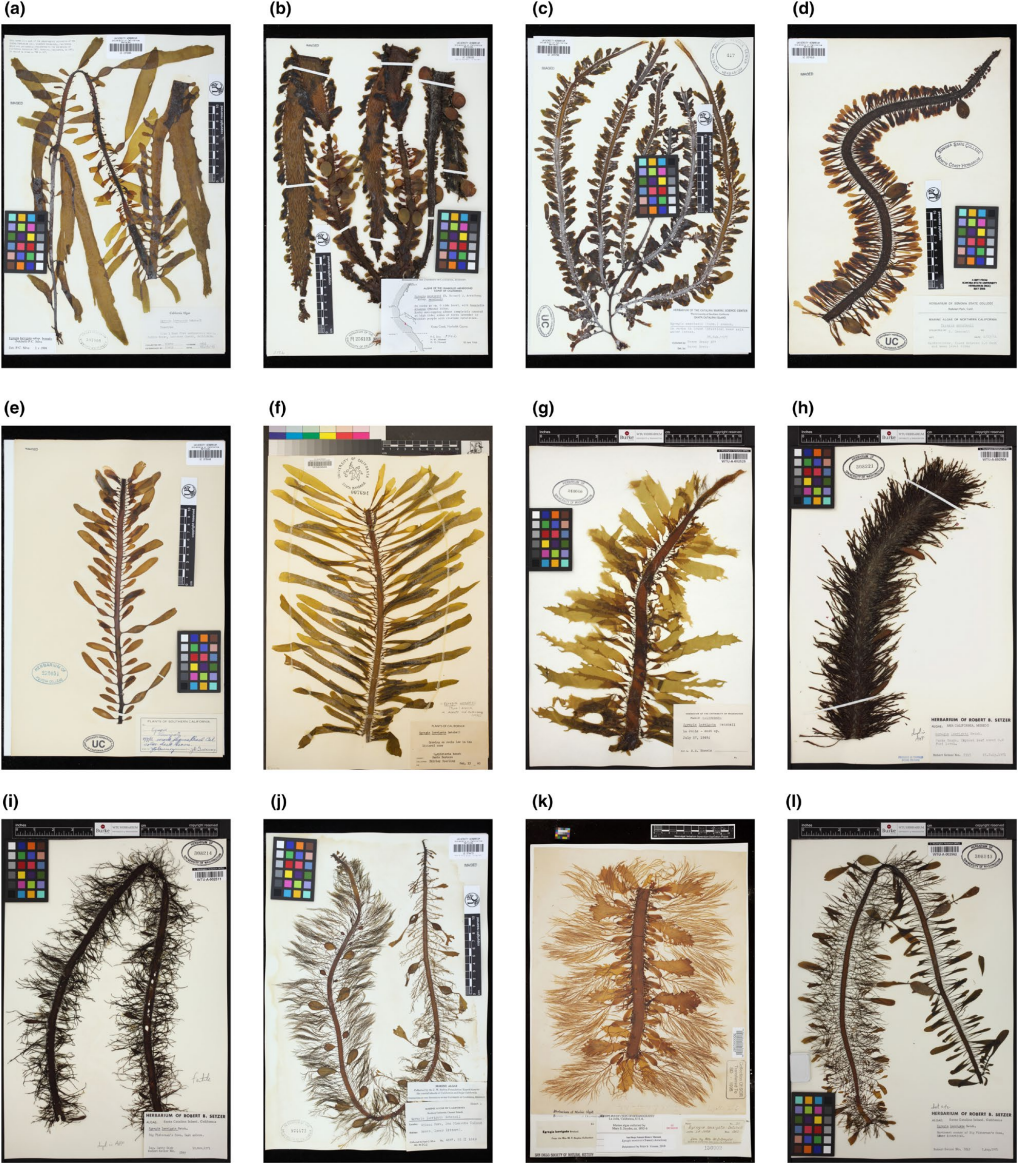
Examples of herbarium specimens of *Egregia menziesii* collected in California with (a) smooth rachis, (b) textured rachis, (c) spatulate blades, (d) clubbed blades, (e) oblong blades, (f) laminar blades, (g) dissected blades, (h) needle‐shaped blades, (i) filiform blades, (j) branched blades, (k) dissected and branched blades, and (l) oblong, filiform, and branched blades. Images are courtesy of the Algae Herbarium Portal and University Herbarium.

For each specimen, we visually characterized the rachis texture (either “smooth” or “textured” with tubercles or papillae; Figure 1a,b), lateral blade shape(s), and the presence or absence of sporophylls. Blade shape categories consisted of “spatulate” (ovate or paddle‐shaped; Figure 1c), “clubbed” (narrow near the rachis and broadening toward the end; Figure 1d), “oblong” (rounded with the same width throughout; Figure 1e), “laminar” (longer than “oblong” and squared off; Figure 1f), “dissected” (with many jagged, sharp, or curved edges; Figure 1g), “needle” (thin and uniform; unbranched; Figure 1h), “filiform” (thread‐like with short, irregular branches; Figure 1i), and “branched” (with multiple orders of branching; Figure 1j). More than one blade shape was usually observed in an individual (Figure 1k,l). To calculate pneumatocyst density, we counted the number of pneumatocysts and measured the overall thallus length (excluding the terminal lamina, which does not bear pneumatocysts) in ImageJ (Schneider et al., 2012) using the ruler in the image for scale. To estimate blade density, we counted the number of lateral blades within a random 2‐cm^2^ swath along the rachis.

### Data analysis

All data visualizations and statistical analyses were conducted in RStudio version 4.3.0 (R Core Team, 2020). We plotted the relative proportions of rachis texture and lateral blade shapes by county latitudinally from north to south. Correlation matrices for blade shape co‐occurrence were visualized using ggcorrplot (Kassambara, 2023). To determine whether blade or pneumatocyst density varied by region or habitat, we ran separate non‐parametric Kruskal–Wallis rank sum tests since group sizes were uneven and residuals were not normally distributed. Kruskal–Wallis tests with Dunn's pairwise comparisons were also used to determine whether trait richness (in terms of the number of unique lateral blade shapes observed by county) varied by region, and whether blade density (number of lateral blades per meter of rachis) varied by blade shape.

We combined the morphological variable groups rachis texture (categorical), lateral blade shape (categorical), and blade or pneumatocyst density (continuous) and constrained them to two dimensions using a multiple factor analysis (MFA, an alternative to a principal component analysis that can handle groups of continuous and categorical variables; Abdi et al., 2013). We then ran a multiple linear regression on the data subset to evaluate the relationship between integrated morphology scores from the first dimension of the MFA and environmental variables (temperature, wave height, or upwelling index, excluding latitude since it is correlated with temperature). For significant predictors, we visualized their effect on rachis texture or blade shape through violin plots. Finally, for both the full dataset and data subset, we incorporated environmental variables into the MFA, calculated eigenvalues, and plotted the individual specimens' coordinates in relation to each of the variables using factoextra (Kassambara & Mundt, 2016) and FactoMineR (Lê et al., 2008). We could not use collection year in our multivariate analysis since there were fewer historical specimens, and the dataset was unbalanced; instead, we grouped all specimens into four time frames (pre‐1900s, 1900–1949, 1950–1999, and 2000–present) and plotted relative proportions of rachis texture and blade shape by region through time.

## RESULTS

**FIGURE 2:**
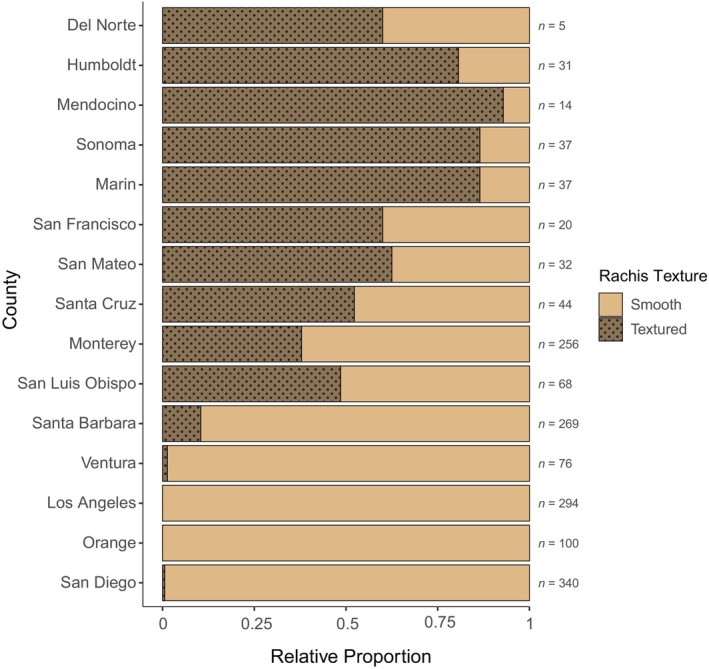
Stacked bar plots showing the relative proportion of rachis texture seen in all herbarium specimens by county in California (with sample sizes on the right), arranged latitudinally from north to south.

**FIGURE 3:**
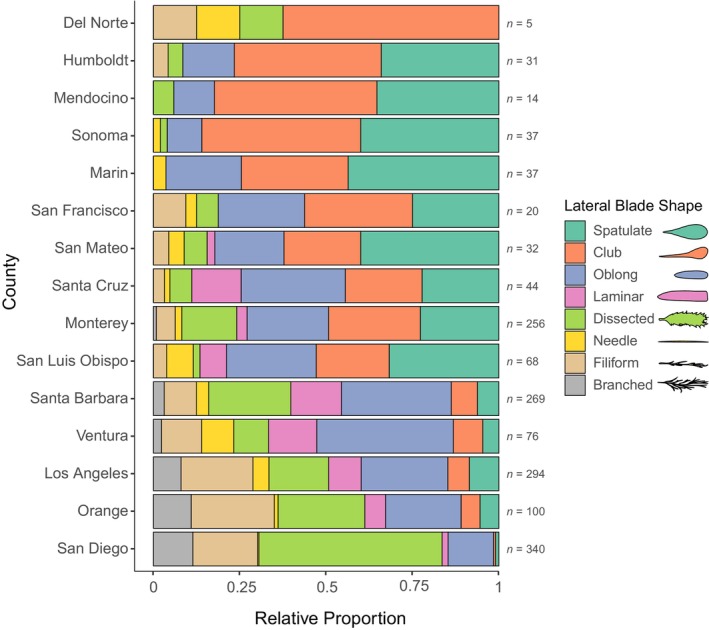
Stacked bar plots showing the relative proportion of lateral blade shapes seen in all herbarium specimens by county in California (with sample sizes on the right), arranged latitudinally from north to south.

**FIGURE 4:**
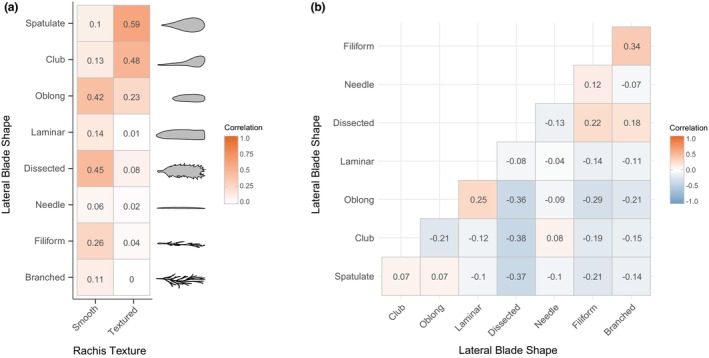
Correlation heatmaps for (a) rachis texture and corresponding lateral blade shape and (b) co‐occurrence of lateral blade shapes, with numbers representing Pearson's correlation coefficient, shaded by magnitude.

Visual characterization of rachis texture across digitized herbarium specimens of *Egregia menziesii* in California over time showed that individuals from northern counties had predominantly textured rachis, those from southern counties had mostly smooth rachis, and central counties had either smooth or textured rachis (Figure 2). Clubbed or spatulate lateral blades were more common in northern and central counties, whereas southern counties had more dissected, filiform, or branched blades (Figure 3). Oblong blades were seen all along the coast, and laminar blades occurred only in central and southern counties, which exhibited significantly greater diversities of blade shapes (Kruskal–Wallis chi‐squared = 12.35, *df* = 2, *p* = 0.002). The presence of a smooth rachis was associated more frequently with dissected, oblong, and/or filiform blades, whereas the presence of a textured rachis was more often associated with clubbed and/or spatulate blades (Figure 4a). Most specimens had more than one lateral blade shape, and certain combinations of blade shapes occurred more often, including oblong and laminar, filiform and branched, dissected and filiform, or branched and dissected (Figure 4b).

**FIGURE 5:**
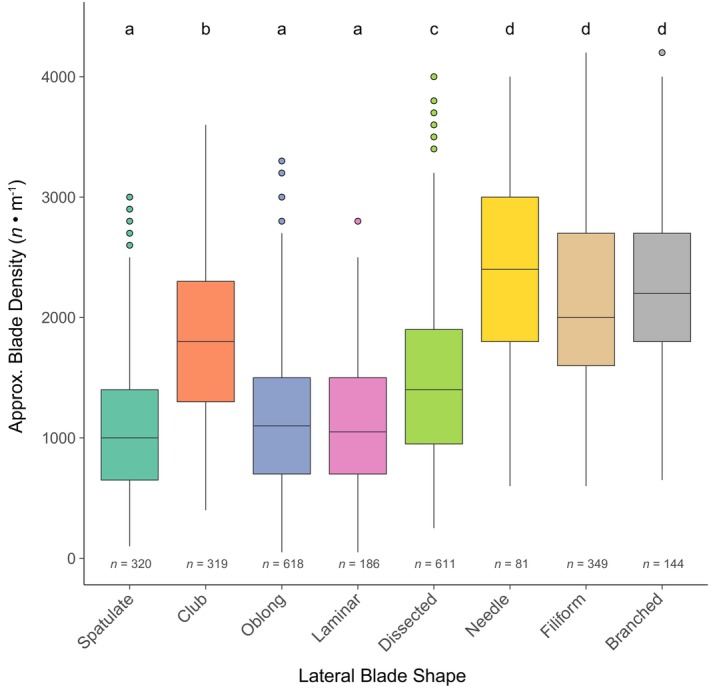
Boxplots for approximate blade density (number of lateral blades per meter of rachis) by blade shape, with sample sizes on the bottom and letters indicating significant groupings.

**FIGURE 6:**
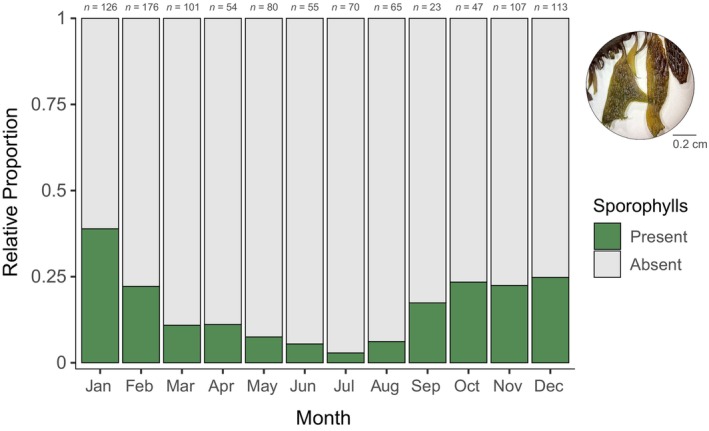
Stacked bar plots showing the proportion of herbarium specimens (with sample sizes on top) in which sporophylls were identified, by month, only for southern California. An example of southern‐type sporophylls is pictured on the top right, with the scale bar representing 0.2 cm.

Lateral blade shapes occurred at significantly different densities (Kruskal–Wallis chi‐squared = 783.45, *df* = 7, *p* < 0.001): Broader shapes such as spatulate, oblong, and laminar generally occurred at a lower density, and narrower shapes such as needle, filiform, and branched occurred at a higher density (Figure 5). There were no significant differences in blade density by region, although blade density was higher on average in the south at 1513 ± 24.1 blades · m^−1^ of rachis (mean ± *SE*) compared with 1424 ± 39.1 and 1444 ± 62.6 blades · m^−1^ in central and northern regions, respectively. Pneumatocyst density was significantly higher in the south at 21.7 ± 0.8 pneumatocysts · m^−1^ compared with 8.8 ± 0.6 and 10.9 ± 1.3 pneumatocysts · m^−1^ in central and northern regions, respectively (Kruskal–Wallis chi‐squared = 111.08, *df* = 2, *p* < 0.001). No significant difference in pneumatocyst density was detected by habitat, although subtidal specimens had more pneumatocysts on average at 19.8 ± 3.4 compared to 14.4 ± 0.7 pneumatocysts · m^−1^ for intertidal specimens. Specimens collected in the drift were excluded from this analysis. Since sporophylls could not be identified in herbarium specimens of northern‐type *Egregia menziesii*, we described reproductive phenology only from southern California. Sporophylls in southern‐type *E. menziesii* (either furrowed with ridges or smooth and dark) were present throughout the year but were most abundant in the fall and winter months from September to February (Figure 6).

**FIGURE 7:**
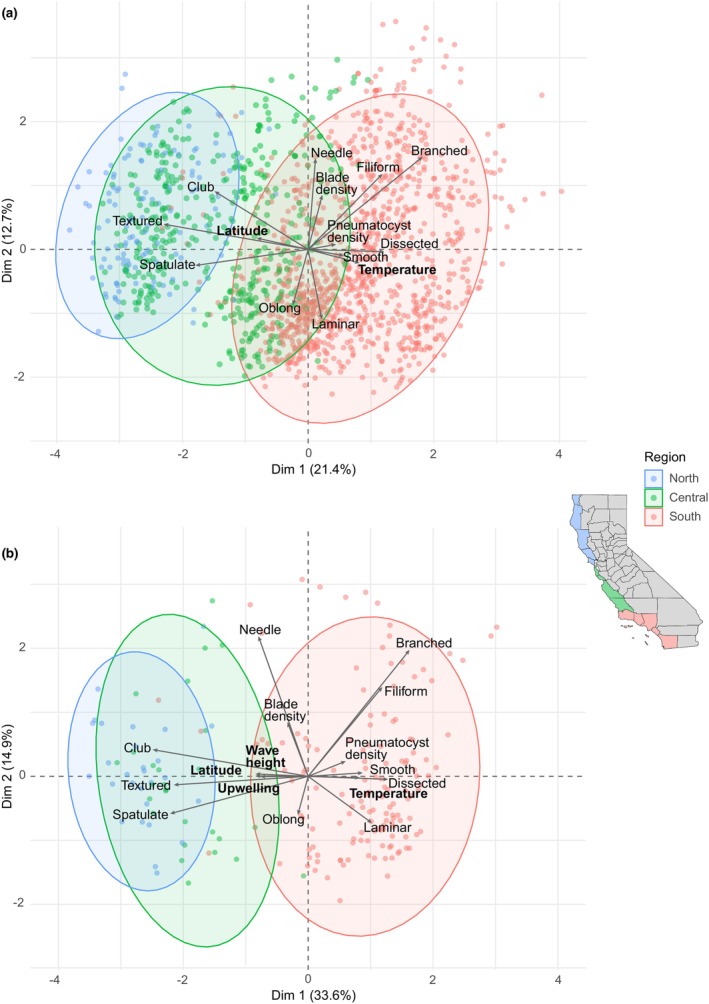
Visualization of dimensions 1 and 2 of a multiple factor analysis incorporating both morphological (rachis texture, lateral blade shape, and blade or pneumatocyst density) and environmental (latitude, temperature, wave height, and upwelling index) variables on (a) the full dataset (1865–present) and (b) the data subset (1989–2019). Each point represents an herbarium specimen, color‐coded by region in California.

**FIGURE 8:**
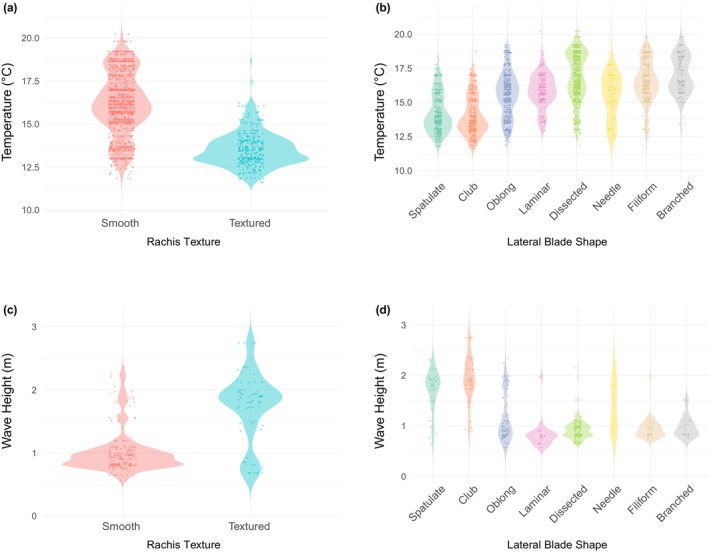
Violin plots (with raw data overlaid) showing the effects of (a) temperature on rachis texture, (b) temperature on lateral blade shape, (c) wave height on rachis texture, and (d) wave height on lateral blade shape.

The MFA visualizations for both the full dataset (*n* = 1599 observations from 1865 to present; Figure 7a) and data subset (*n* = 194 observations from 1989 to 2019; Figure 7b) had similar clusters by region, with central overlapping north and south. Higher latitudes were characterized by textured rachis and clubbed or spatulate blades, whereas lower latitudes were characterized by smooth rachis and a variety of blade shapes as well as higher blade and pneumatocyst density. When considering just latitude and temperature as environmental variables, only 34.2% of variance was explained overall in the first two dimensions of the MFA (Table S1), and time frame explained the most variance in subsequent dimensions (Table S2). When also considering wave height and upwelling index, 48.6% of variance was explained in the first two MFA dimensions (Table S3). Temperature and wave height were significant predictors of morphology (*p* < 0.001; Table S4, Figure S1) with estimated effect sizes of −0.26 ± 0.05 and 1.41 ± 0.16, respectively. Specifically, textured rachis occurred mostly at lower temperatures or higher wave heights whereas smooth rachis occurred across all temperatures and mostly lower wave heights (Figure 8a,c). Spatulate or clubbed blades were more common at lower temperatures and higher wave heights whereas dissected, filiform, or branched blades were more common at higher temperatures and lower wave heights (Figure 8b,d). Morphological characteristics by region were largely consistent across time frames, although from 2000 to present, *Egregia menziesii* rachis in central California became more textured (Figure S2) and lateral blades in southern California became relatively more dissected (Figure S3).

## DISCUSSION

Our herbarium‐based morphological characterization of *Egregia menziesii* aligns with and expands upon the characterizations reported in the literature (Abbott & Hollenberg, 1976; Blanchette et al., 2002; Chapman, 1961; Henkel et al., 2007; Setchell & Gardner, 1925; Silva, 1957). In our study, morphological variation along latitudes was related to environmental factors such as SST and wave height but not coastal upwelling index. Patterns in rachis texture and lateral blade shape by region showed a gradient in characteristics from north to south with gradual replacement. Although we did not detect major morphological differences by time frame, the correlation between morphology and temperature raises the question of whether in the future, rising seawater temperatures due to climate change will lead to the occurrence of more southern‐type *E. menziesii* farther north. However, given that wave height had a stronger effect on morphology than temperature, even if we expect southern‐type *E. menziesii* to shift northward, they may not be as successful in higher wave‐energy environments.

Environmental or biological factors that could not be directly tied to historical specimens but that may have affected the morphology of *Egregia menziesii* include nutrient availability (Blanchette et al., 2002; Kraemer & Chapman, 1991b), seasonality (Burnett & Koehl, 2019), desiccation stress (Hughes, 2010), and grazing (Black, 1974; Bracken & Stachowicz, 2007). For example, limpets associated with *E. menziesii* can cause sublethal tissue loss, frond breakage, or thallus mortality (Black, 1976; Burnett & Koehl, 2020), and textured rachis are, perhaps, less susceptible to mechanical damage. Similarly, rachis with denser papillae can retain more water and better withstand desiccation in the intertidal zones (Fulton‐Bennett, 2016). At higher wave‐energy sites, where juveniles of both northern and southern morphotypes are present, lower adult survivorship has been seen for thalli with smooth rachis, and vice versa (Henkel et al., 2007). Additional factors that could promote morphological plasticity are genetics (Henkel et al., 2007; Lane et al., 2006), local acclimation (Henkel & Hofmann, 2008), and site or habitat‐specific differences (Burnett & Koehl, 2019; Fulton‐Bennett, 2016), although it could be argued that the morphology of *E. menziesii* is more of a response to environmental conditions differentially selecting for genetically determined traits (Henkel et al., 2007).

Notably, we utilized more categories for lateral blade shape than Henkel et al. (2007) and recorded instances of multiple blade shapes in a single specimen, which allowed us to highlight morphological variability not only between but also within individuals. Our observation that blade density varied by shape was not surprising, since narrower blades can be packed more tightly and have a lower surface area‐to‐volume ratio; as such, more blades would be needed to efficiently take up nutrients (Littler, 1980; Odum et al., 1958; Stewart & Carpenter, 2003). Since waters off southern California are more nutrient‐limited than those to the north, we had expected blade density to vary by region, but our results were not statistically significant. Still, the greater diversity of blade shapes in southern morphotypes suggests that this could be an adaptation to nutrient limitation. Additional blade functional traits (length, width, perimeter, surface area, circularity, etc.) that are continuous rather than categorical would enhance these morphometrics (Fong et al., 2023) but would require destructive sampling, as blades in *Egregia menziesii* often overlap. Particularly for dissected blades, the degree of dissection was once thought to be indicative of developmental stage (Chapman, 1961), yet new *E. menziesii* blades are formed in between older blades on the rachis margins throughout the lifetime of the thallus. It would be interesting to tag and track differences in morphology during the formation of a subset of blades along the same section of the rachis on a live individual to see whether differences reflect environmental variation on shorter time scales.

The higher pneumatocyst density in southern‐region *Egregia menziesii*, which generally experience lower wave energy than in northern California, contradicted results from a past study in which *E. menziesii* at more wave‐exposed sites had larger and more numerous pneumatocysts (Burnett & Koehl, 2017). We did not measure pneumatocyst size, which could account for differences in density; perhaps northern forms have fewer yet larger, more buoyant pneumatocysts. Although not statistically significant, the higher pneumatocyst density in subtidal specimens corroborated observations by Chapman (1961) on differences between shallow and deep‐water *E. menziesii*, but further study of pneumatocyst size and shape by habitat or region is warranted. It is also worth mentioning that our specimens were not all taken from the same part of the individual (e.g., adjacent to or at a certain distance from the meristem), and there is considerable variation along the length of these perennial fronds; thus, our results may not represent full thallus morphology. For example, differences in the orders of thallus branching among northern and southern morphotypes have previously been noted (Blanchette et al., 2002), as well as thalli with both smooth and textured rachis on the same individual (Henkel et al., 2007). However, we could not reliably assess this aspect of morphology from herbarium specimens with partial thalli.

Little is known about the reproductive phenology of *Egregia menziesii* (but see Myers, 1928), and our results were consistent with field observations by Henkel and Murray (2007) that sporophyll production in southern California populations peaks in the winter and drops in the spring and summer. This conflicts with the statement by Abbott and Hollenberg (1976) that sporophylls were most abundant between April and November, although they may have been referring to northern populations. It was difficult to distinguish sporophylls from vegetative blades in northern‐type *E. menziesii* since they were less conspicuous by eye (Adi Khen, pers. obs), let alone in digitized herbarium specimens. Repeated monitoring surveys or targeted collections tracking sporophyll presence or absence at the same locations by month in situ would complement our herbarium‐derived observations. Unfortunately, we could not make inferences about monthly sporophyll presence across time frames due to insufficient sample sizes, but future studies should examine whether the timing of sporophyll production in *E. menziesii* has changed under altered seasonal temperature regimes.

Analyses of macroalgal herbarium specimens have been used to study climate‐driven morphological responses in species from the Gelidiales (Alfonso et al., 2022) and Fucales (Geppi & Riera, 2022). In conjunction with contemporary specimens, historical specimens can reveal species range shifts (Riera et al., 2015, Tingley & Beissinger, 2009) or invasion history (Miller et al., 2011; Steen et al., 2017). Dried tissue samples from herbarium specimens have also been used to quantify stables isotopes of carbon and nitrogen, to reconstruct historical nutrient levels (Alldred et al., 2024; Miller et al., 2020). Although we did not see an effect of upwelling (as a proxy for nutrients) on morphology, perhaps there would have been a correlation if we had used chemical signatures obtained from the specimens themselves. Additionally, as more collections are digitized, the capacity and accessibility for scientific research from herbaria is expanding (Heberling & Isaac, 2017; Hedrick et al., 2020; Soltis, 2017). For example, herbarium specimens will potentially be useful for exploring the “tropicalization” of temperate ecosystems due to climate change. Since DNA has successfully been extracted from herbarium samples over a century old (Hughey et al., 2024; Provan et al., 2008; Schipper et al., 2023), many of these specimens could be important for revisiting taxonomy and/or phylogenetics of key taxa.

Some limitations to the use of herbaria for research are that they are prone to sampling biases (e.g., inconsistent collection efforts that do not represent all taxa or locations), and collection details can be sparse or incomplete. Specimens are chosen or modified to fit on herbarium paper, and collectors often choose a specimen as a voucher for its occurrence rather than to demonstrate individual variation. Specimens of *Egregia menziesii*, with flat axes and marginal blades, were amenable to our herbarium‐based morphological analyses but specimens with complex, radial branching would be more challenging. Nevertheless, herbaria are not just inherently valuable for the sake of preserving natural history; they can also inform ecologically relevant questions on taxonomy, distribution, biodiversity, form and function, or phenology. Our results validated field‐based observations while providing additional insights, which ultimately speaks to the value of historical and contemporary collections. This serves as a case study for using digitized herbarium specimens to characterize morphological variability across space and over time, which could be applied to other taxa of interest.

## AUTHOR CONTRIBUTIONS

**Adi Khen:** Conceptualization (equal); data curation (lead); formal analysis (lead); visualization (lead); writing – original draft (lead). **Kai M. Moore:** Data curation (equal); visualization (supporting); writing – review and editing (supporting). **Siobhan A. Braybrook:** Conceptualization (equal); formal analysis (supporting); writing – review and editing (equal). **Peter S. Vroom:** Data curation (supporting); writing – review and editing (supporting). **Kathy Ann Miller:** Conceptualization (equal); data curation (supporting); writing – review and editing (equal). **Jennifer E. Smith:** Conceptualization (equal); formal analysis (supporting); supervision (lead); visualization (supporting); writing – review and editing (equal).

## FUNDING INFORMATION

Funding was provided by the Beyster Family, Ellen Browning Scripps Family Foundation, and the California Institute for Biodiversity.

## Supporting information


**Figure S1.** Pearson's correlations between multiple factor analysis dimension 1 scores using only morphological variables and (a) temperature, on the full dataset or (b) wave height, on the data subset. Each point represents an herbarium specimen, color‐coded by region in California.

**Figure S2.** Stacked bar plots showing the relative proportion of rachis texture seen in all herbarium specimens, by time frame and region in California (with sample sizes on the right).

**Figure S3.** Stacked bar plots showing the relative proportion of lateral blade shapes seen in all herbarium specimens, by time frame and region in California (with sample sizes on the right).

**Table S1.** Eigenvalues and proportions of explained variance for the first five dimensions of the multiple factor analysis on the full dataset incorporating morphological, environmental (only latitude and temperature), and temporal variables.

**Table S2.** Contributions of groups to the first five dimensions of the multiple factor analysis on the full dataset incorporating morphological, environmental (only latitude and temperature), and temporal variables.

**Table S3.** Eigenvalues and proportions of explained variance for the first five dimensions of the multiple factor analysis on the data subset incorporating morphological and all environmental variables (latitude, temperature, wave height, and upwelling index).

**Table S4.** Statistical output from a multiple linear regression testing for the effects of environmental variables on morphology for the data subset. Bold indicates statistical significance (α = 0.05).

## Data Availability

Compiled herbarium metadata and morphometrics collected for this study, along with R codes for data visualization and statistical analysis, are available at https://github.com/akhen1/egregia‐morphology.
