# Supplementary figures and images for: Characterizing morphology of Egregia menziesii (Laminariales) in California over 2 centuries using historical and contemporary herbarium specimens
Source: J Phycol. 2026 Jan 20;62(1):82–95. doi: 10.1111/jpy.70126 (PMC12961177; doi:10.1111/jpy.70126)

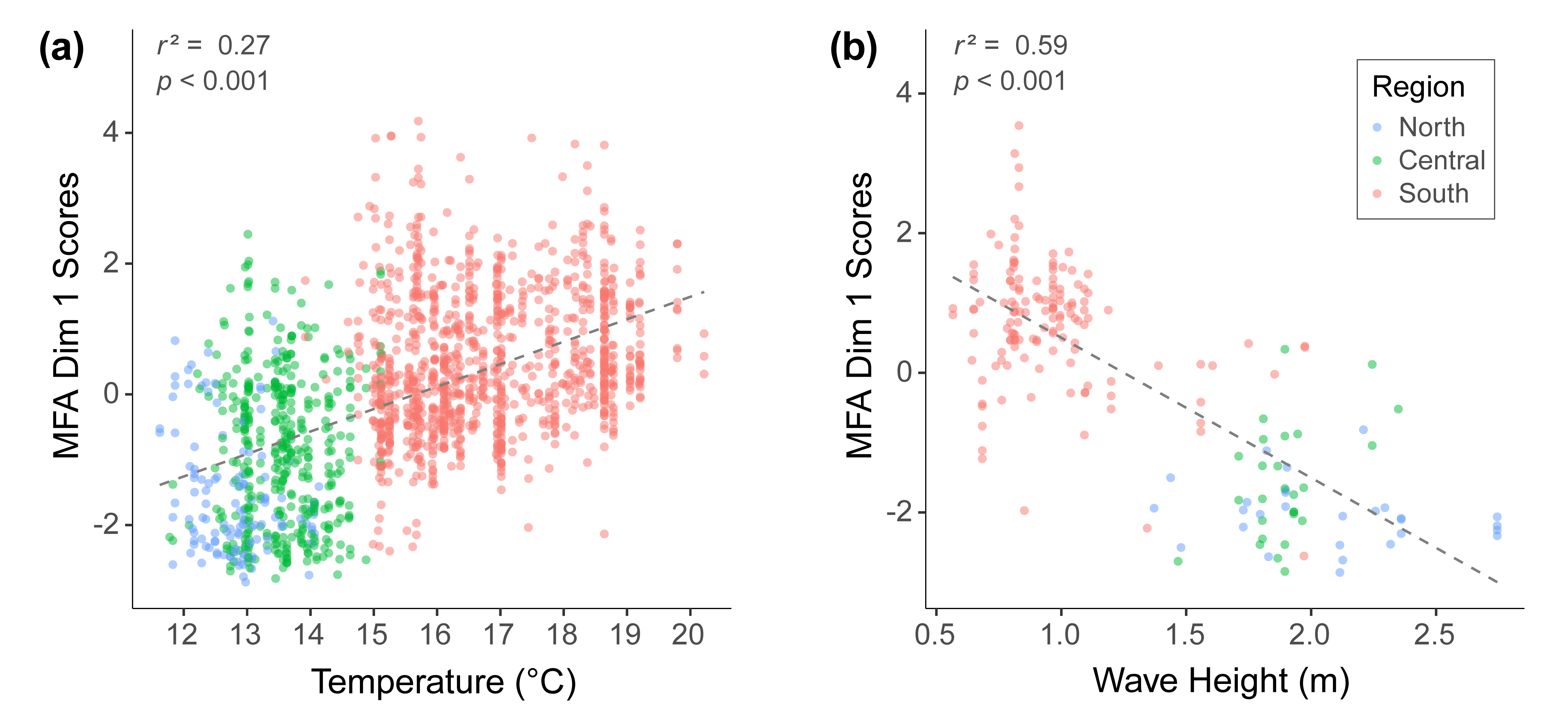

Supplement: Supplementary file 1 — Figure S1. Pearson's correlations between multiple factor analysis dimension 1 scores using only morphological variables and (a) temperature, on the full dataset or (b) wave height, on the data subset. Each point represents an herbarium specimen, color‐coded by region in California. [file JPY-62-82-s004.tif]

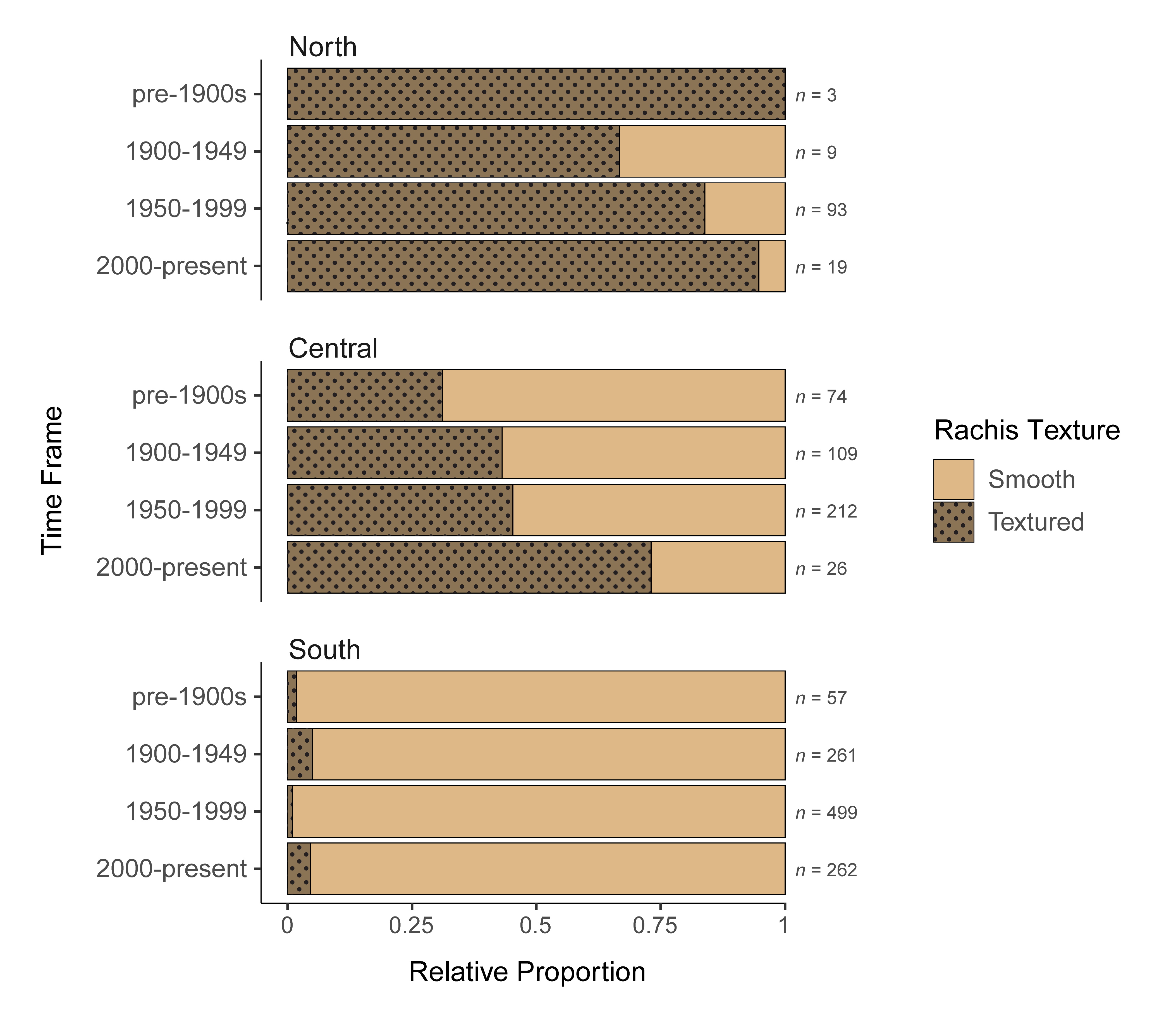

Supplement: Supplementary file 2 — Figure S2. Stacked bar plots showing the relative proportion of rachis texture seen in all herbarium specimens, by time frame and region in California (with sample sizes on the right). [file JPY-62-82-s005.tif]

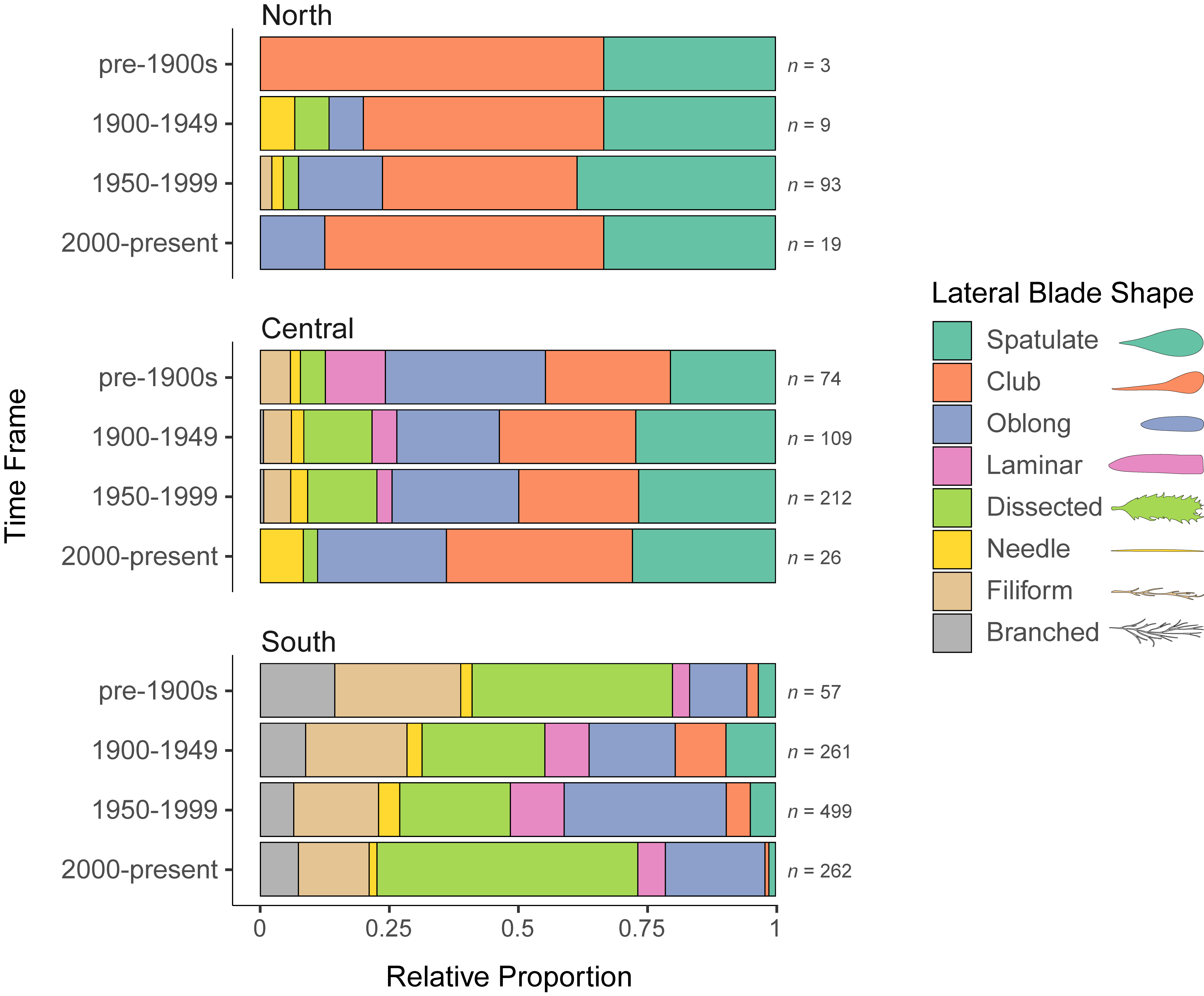

Supplement: Supplementary file 3 — Figure S3. Stacked bar plots showing the relative proportion of lateral blade shapes seen in all herbarium specimens, by time frame and region in California (with sample sizes on the right). [file JPY-62-82-s002.tif]
