# Supplementary material for: Characterizing morphology of Egregia menziesii (Laminariales) in California over 2 centuries using historical and contemporary herbarium specimens
Source: J Phycol. 2026 Jan 20;62(1):82–95. doi: 10.1111/jpy.70126 (PMC12961177; doi:10.1111/jpy.70126)
Supplement: Supplementary file 4 — Table S1. Eigenvalues and proportions of explained variance for the first five dimensions of the multiple factor analysis on the full dataset incorporating morphological, environmental (only latitude and temperature), and temporal variables. [file JPY-62-82-s006.docx]

**Table S1:** Eigenvalues and proportions of explained variance for the first five dimensions of the multiple factor analysis on the full dataset incorporating morphological, environmental (only latitude and temperature), and temporal variables.

|  | **Eigenvalue** | **% Variance Explained** | **Cumulative % Variance** |
| --- | --- | --- | --- |
| Dim 1 | 2.31 | 21.43 | 21.43 |
| Dim 2 | 1.37 | 12.72 | 34.15 |
| Dim 3 | 1.13 | 10.47 | 44.62 |
| Dim 4 | 1.03 | 9.54 | 54.16 |
| Dim 5 | 1.00 | 9.29 | 63.45 |
