# Supplementary material for: Characterizing morphology of Egregia menziesii (Laminariales) in California over 2 centuries using historical and contemporary herbarium specimens
Source: J Phycol. 2026 Jan 20;62(1):82–95. doi: 10.1111/jpy.70126 (PMC12961177; doi:10.1111/jpy.70126)
Supplement: Supplementary file 5 — Table S2. Contributions of groups to the first five dimensions of the multiple factor analysis on the full dataset incorporating morphological, environmental (only latitude and temperature), and temporal variables. [file JPY-62-82-s007.docx]

**Table S2:** Contributions of groups to the first five dimensions of the multiple factor analysis on the full dataset incorporating morphological, environmental (only latitude and temperature), and temporal variables.

| **Variable Group** | **Dim 1** | **Dim 2** | **Dim 3** | **Dim 4** | **Dim 5** |
| --- | --- | --- | --- | --- | --- |
| Latitude, temperature (continuous) | 31.14 | 3.02 | 1.61 | 1.09 | 0.44 |
| Lateral blade shape (categorical) | 26.66 | 39.43 | 12.22 | 3.72 | 3.31 |
| Rachis texture  (categorical) | 24.49 | 2.05 | 15.36 | 6.05 | 0.02 |
| Blade/pneumatocyst density (continuous) | 9.96 | 49.30 | 18.14 | 2.20 | 4.12 |
| Time frame  (categorical) | 7.74 | 6.20 | 52.67 | 86.93 | 92.11 |
