# Supplementary material for: Characterizing morphology of Egregia menziesii (Laminariales) in California over 2 centuries using historical and contemporary herbarium specimens
Source: J Phycol. 2026 Jan 20;62(1):82–95. doi: 10.1111/jpy.70126 (PMC12961177; doi:10.1111/jpy.70126)
Supplement: Supplementary file 6 — Table S3. Eigenvalues and proportions of explained variance for the first five dimensions of the multiple factor analysis on the data subset incorporating morphological and all environmental variables (latitude, temperature, wave height, and upwelling index). [file JPY-62-82-s001.docx]

**Table S3:** Eigenvalues and proportions of explained variance for the first five dimensions of the multiple factor analysis on the data subset incorporating morphological and all environmental variables (latitude, temperature, wave height, and upwelling index).

|  | **Eigenvalue** | **% Variance Explained** | **Cumulative % Variance** |
| --- | --- | --- | --- |
| Dim 1 | 2.75 | 33.61 | 33.61 |
| Dim 2 | 1.22 | 14.94 | 48.56 |
| Dim 3 | 0.91 | 11.08 | 59.64 |
| Dim 4 | 0.64 | 7.82 | 67.46 |
| Dim 5 | 0.55 | 6.66 | 74.11 |
