# Supplementary material for: Characterizing morphology of Egregia menziesii (Laminariales) in California over 2 centuries using historical and contemporary herbarium specimens
Source: J Phycol. 2026 Jan 20;62(1):82–95. doi: 10.1111/jpy.70126 (PMC12961177; doi:10.1111/jpy.70126)
Supplement: Supplementary file 7 — Table S4. Statistical output from a multiple linear regression testing for the effects of environmental variables on morphology for the data subset. Bold indicates statistical significance (α = 0.05). [file JPY-62-82-s003.docx]

**Table S4:** Statistical output from a multiple linear regression testing for the effects of environmental variables on morphology for the data subset. Bold indicates statistical significance (𝛼 = 0.05).

| **Predictor** | **Estimate** | **Std. Error** | ***t* value** | ***p*-value** |
| --- | --- | --- | --- | --- |
| (Intercept) | 2.641 | 0.915 | 2.886 | **0.004** |
| Temperature | -0.264 | 0.047 | -5.553 | **<0.001** |
| Upwelling | -0.049 | 0.046 | -1.072 | 0.285 |
| Wave height | 1.408 | 0.162 | 8.705 | **<0.001** |
